# Identification of 2-Cys Peroxiredoxin (BmTPx-2) as Antioxidant Active Molecule from *Babesia microti*

**DOI:** 10.3389/fmicb.2017.01959

**Published:** 2017-10-10

**Authors:** Xunan Hai, Houshuang Zhang, Zhonghua Wang, Haiyan Gong, Jie Cao, Yongzhi Zhou, Jinlin Zhou

**Affiliations:** ^1^Key Laboratory of Animal Parasitology of Ministry of Agriculture, Shanghai Veterinary Research Institute, Chinese Academy of Agricultural Sciences, Shanghai, China; ^2^Jiangsu Co-innovation Center for Prevention and Control of Important Animal Infectious Diseases and Zoonoses, Yangzhou, China

**Keywords:** *Babesia microti*, peroxiredoxin, identification, activity assay, transcription analysis

## Abstract

Peroxiredoxins (Prxs) are a family of antioxidant enzymes that reduce peroxides in the presence of thioredoxin, thioredoxin reductase, and nicotinamide adenine dinucleotide phosphate (NADPH) to resist oxidative stress. In this study, we identified and isolated a 2-Cys Prx designated as ‘*BmTPx-2*’ from *Babesia microti*, with a full-length cDNA of 826 bp and an open reading frame of 756 bp, which encodes a 251-amino acid protein. BLAST analysis demonstrated that BmTPx-2 shows the typical features of members of the 2-Cys Prx family, which includes harboring two conserved VCP motifs with Cys^101^ and Cys^221^ conserved cysteine residues. Recombinant BmTPx-2 was expressed in *Escherichia coli* and analyzed by western blot. The antioxidant activity of BmTPx-2 was demonstrated using a mixed-function oxidation system and oxidation of NADPH. Furthermore, *BmTPx-2* mRNA expression level in parasites at the erythrocytes and tick stages were analyzed by real-time fluorescence quantitative PCR. Peak *BmTPx-2* mRNA transcription was detected 8 days after infection at the erythrocyte stage, but not at the tick stage. Taken together, this study characterized *BmTPx-2* from *B. microti* as an antioxidant molecule that was specifically transcribed at the erythrocyte stage.

## Introduction

*Babesia* species belong to phylum Apicomplexa, which mainly comprises intra-erythrocytic protozoan parasites (Vannier et al., 2015). *Babesia* is transmitted by tick bites, blood transfusions, and transplacentally. *Babesia* was first described in 1888; 5 years later, the tick was confirmed as its transmission vector, and in 1957, human babesiosis was identified (Vannier et al., 2008, 2015). *Babesia* consists of 100s of species, and among these, *Babesia microti* is the most frequent cause of human and rodent babesiosis. Human babesiosis has been reported in China (Vannier et al., 2008; Zhou et al., 2013, 2015). In 2012, the entire genome of *B. microti* was sequenced and mapped (Cornillot et al., 2012, 2013). *B. microti* causes red blood cell lysis, jaundice, hemolytic anemia, remittent fever, and renal insufficiency, and mortality rate is estimated at 10% following *B. microti* infection (White et al., 1998; Hatcher et al., 2001; Zhou et al., 2015). Moreover, the fatality rate is higher among those who are immunocompromised or acquire the infection through blood transfusion (Krause et al., 2008; Herwaldt et al., 2011; Leiby, 2011). *B. microti* has been recognized as an emerging worldwide health threat; therefore, the identification of an efficient and safe method to prevent and treat babesiosis is imperative (Vannier et al., 2008, 2015; Zhou et al., 2013).

*Babesia microti* lives in oxygen-rich erythrocytes that generate abundant reactive oxygen species (ROS), which damage biological macromolecules such as nucleic acids and proteins of *B. microti*, and to counter this effect, the parasite is equipped with anti-oxidation molecules such as thioredoxin, peroxiredoxins (Prxs), glutaredoxins, and superoxide dismutases. Prxs play an important roles against the toxicity of ROS, which are generated during hemoglobin digestion (Storz and Imlay, 1999; Tanaka et al., 2009; Robinson et al., 2010; Masatani et al., 2014, 2016; Wang et al., 2016). Prxs were initially discovered in 1989 in *S. typhimurium*, with an ability to resolve organic peroxide cumene hydroperoxide. Prxs play an array of functions, and a few have been confirmed in parasites, and the most relevant involves its thioredoxin-dependent peroxidase activity (Rhee and Woo, 2011; Gretes et al., 2012; Angelucci et al., 2016). The reduction of Prx expression by knockout (KO) and RNA interference (RNAi) is lethal to the survival of the parasite, thus confirming that Prxs are crucial to their survival and virulence (Sayed et al., 2006; Castro et al., 2011; Kimura et al., 2013; Hakimi et al., 2014; Usui et al., 2015). Recent studies have demonstrated that the physiological roles of parasite Prxs involve their potential use chemotherapy and as drug targets (Ginsburg et al., 1999; Krauth-Siegel et al., 2005).

Prxs are divided into three types based on the number and position of conserved cysteine residues, namely, 1-Cys, typical 2-Cys and 2-Cys type (Rhee et al., 2001; Wood et al., 2003; Rhee, 2016). The functional unit of typical 2-Cys Prxs is a homodimer (Angelucci et al., 2015, 2016). However, the 2-Cys TPx-2 of *B. microti* has not been characterized to date. Therefore, the main focus of the present study was to identify and to elucidate the function of the antioxidant biological properties of TPx-2, a novel Prx of *B. microti.* In this study, we obtained a novel Prx protein from *B. microti*, which we called ‘BmTPx-2.’ BLASTp analysis determined that BmTPx-2 belongs to the thioredoxin-like superfamily. *BmTPx-2* is a typical 2-Cys Prx, and amino acid sequence alignment with other 2-Cys Prx indicated that BmTPx-2 also harbored two conserved cysteine residues (Cys^101^ and Cys^221^), in which Cys^101^ was a peroxidatic acid and Cys^221^ was a resolving acid.

## Materials and Methods

### Parasite Culture

The *B. microti* strain was obtained from the American Type Culture Collection (ATCC, PRA-99^TM^) and was injected into Kunming mice. *B. microti* was maintained in purified mice erythrocytes and the parasite was isolated when the erythrocyte infection rate reached 30–40%, which was confirmed with Giemsa-stained thin-blood smears.

### *B. microti* cDNA Library Construction and Illumina Sequencing

Total RNA of *B. microti* was extracted using TRIzol reagent (Life Technologies Corporation, Carlsbad, CA, United States) and dissolved in RNase-free water. RNA integrity was verified by gel electrophoresis, and quantity was determined using a NanoVue spectrophotometer (GE Healthcare Life Sciences, Pittsburg, PA, United States). cDNA library construction and Illumina sequencing of the samples were performed at Genomics Institute (BGI, Shenzhen, China). RNA sequence libraries were constructed according to Illumina manufacturer’s instructions for 150-bp paired-ends and then sequenced using Illumina HiSeq 2000 (Illumina, United States). A total of 3,024 genes were obtained from transcriptome assembly.

### Molecular Cloning of *BmTPx-2* and Multiple Sequence Alignment Analysis

Based on the open reading frame (ORF) sequence of *BmTPx-2* downloaded from GenBank (XM_012792398.1), we designed a pair of specific primers: BmTPx-2-F: 5′-TT CCATGG CA ATG CGG TAC ATA ACA CTT TTA-3′, BmTPx-2-R: 5′-TT CTCGAG AGA AGT TTG GCC AAA AGC CTC-3′, *Nco*I and *Xho*I sites are underlined. We amplified the ORF full sequence using the cDNA library as template. Then, the purified PCR products were ligated onto a pMD-18T vector (TaKaRa, Japan) and sequenced. Multiple sequence alignment of *BmTPx-2* was performed with 2-Cys TPx-2 of *Plasmodium falciparum* (Pf: GenBank Accession number BAA97121.1), 2-Cys TPx-2 of *Theileria parva* (Tp: XP_765704.1), and 2-Cys TPx-2 of *Tamarix hispida* (Th: FH74407.1), TPx-2 of *Toxoplasma gondii* (Tg: AG25678.2).

### Full-Length Sequence of *BmTPx-2*

To obtain the full sequence of the 3′ full sequence and 5′ full sequence of *BmTPx-2*, the 5′ and 3′ regions of the mRNA were amplified using a SMARTER RACE cDNA amplification kit (Clontech, San Jose, CA, United States), following the manufacturer’s instructions. The primers were designed based on the ORF sequence earlier obtained. The primer sequences were as follows: GSP1: 5′-GACAGCCATGTCACCTCCCT-3′, GSP2: 5′-CAAGTATGTGCCGTATATTTCCAAA-3′, and GSP3: 5′-GCGAGTTTGTAAAGTGAATGTATGG-3′ for 5′ rapid amplification of cDNA ends (RACE); GSP1: 5′-TGGGTAGGAGTGTGGATGAGAC-3′ and GSP2: 5′-AAGGGTAATAAGGGAATGGCGGC-3′ for 3′ RACE. The resulting PCR products were subsequently purified and ligated onto the pMD-18T vector (TaKaRa, Japan) and sequenced. RACE analyses were performed according to the manufacturer’s recommendations for using the 3′ RACE system for rapid amplification of cDNA ends and 5′ RACE system for rapid amplification of cDNA Ends (Invitrogen, United States).

### Expression and Purification of Recombinant Protein BmTPx-2

We cloned the ORF of *BmTPx-2* into the prokaryotic expression vector pET-28a. Then, to generate the fusion protein, the recombinant vector pET-28a-BmTPx-2 was transformed into *Escherichia coli* BL21 cells at 16°C by 1 mM isopropylthio-β-D-galactoside (IPTG) induction. The fusion protein was purified with NI-NTA agarose beads under native conditions (Merck, United States).

### Antioxidant Activity Assay

Next, to evaluate the antioxidant activity of the recombinant proteins, a mixed-function oxidation (MFO) assay was performed to determine the ability of BmTPx-2 in protecting DNA from damage in oxidative conditions: the MFO mix reaction buffer contained 8 mM FeCl_3_, 40 mM dithiothreitol (DTT), 20 mM EDTA, 25 mM HEPES (pH 7), 180 ng pBluescript plasmid DNA, and various concentrations of the purification fusion protein rBmTPx-2 (335, 167, 83, and 41 μg/mL). After mixing, the reaction buffer was pre-incubated at 37°C for 1 h, 180 ng pBluescript plasmid DNA was then added, and the mixture was incubated at 37°C for another 3 h. Nicking of the supercoiled plasmids was detected by 1% agarose gel electrophoresis and stained with ethidium bromide.

A standard experiment to identify the antioxidant activity of rBmTPx-2 was performed in a 200-μL mixture buffer that contained 375 μM of NADPH, 1 mM of H_2_O_2_, 6.4 μM Trx, 0.14 μM TrxR, HEPES-NaOH (pH 7), and 100 μg/mL rBmTPx-2. The negative control group contained H_2_O instead of rBmTPx-2 in the assay system. NADPH oxidation was monitored for 30 min at 30°C by measuring the absorbance at a wavelength of 340 nm using a spectra Max M5 (MD, United States).

### Production of Mouse Antiserum against the Recombinant Protein

Eight-week-old male mice (Slac, China) were used for the production of antiserum against rBmTPx-2. For primary immunization, a mixture of 100 μg of BmTPx-2 and an equal volume of Freund’s complete adjuvant (Sigma, United States) was intraperitoneally injected into the mice. After 2 weeks, boost immunization was performed in a similar manner as the primary immunization. Serum was collected 20 days after boost immunization. All animal experimental procedures were conducted in accordance with the Guiding Principles of Animal Experimentation in the Shanghai Veterinary Research Institute.

### Western Blot Analysis of the Native Protein of Parasites

*Babesia microti*-infected RBCs were collected when the infection reached 40–50%. RBCs were pelleted by centrifugation and hemolyzed with a red cell lysis buffer (TianGen, China), shaken repeatedly, and incubated at room temperature for 5 min. After centrifugation, the pellet was washed thrice with PBS until the supernatant was clear, and freeze-thaw cycles in liquid nitrogen and water bath were performed thrice. The RBC lysate was mixed with an equal volume of SDS-PAGE loading buffer and heated to 100°C for 10 min, and the protein was separated by SDS-PAGE (15% gel) and subsequently transferred onto a polyvinylidene difluoride membrane (Immobilon; Millipore). The membranes were blocked with 5% skim milk diluted in PBS containing 0.05% Tween (PBST) for 2 h at 37°C, and then was washed with 1× PBST thrice. The membranes were reacted with anti-BmTPx-2 mouse serum at 1:100 at 37°C for 1 h, followed by goat anti-mouse IgG (1:2,000). The signals were developed with an Immobilon western chemiluminescence HRP substrate.

### Transcription Analysis in the Erythrocyte and Tick Stages by Real-time PCR

To examine the expression of BmTPx-2 at different stages of infection, 30 6-week-old male mice (Slac, China) were injected with RBCs infected with *B. microti*, and then the mice RBCs were detected by quantitative real-time PCR (qRT-PCR) using the expression of *actin* mRNA as internal control. We divided 30 mice into 10 groups following *B. microti* injection, and blood was collected from three mice/group from the 2nd–11th day after injection. Total RNA of *B. microti* was extracted using the TRIzol method, and cDNA was synthesized according to the PrimeScript RT reagent kit with gDNA eraser (TaKaRa, Japan). qRT-PCR was performed using cDNA as template and a pair of specific primers, following the recommendations provided in the SYBR premix ExTaq (TaKaRa, Japan) with StepOnePlus PCR system. The experimental values were expressed as relative amounts, and analysis was performed with the 2^-ΔΔ*c*_t_^ method.

To detect BmTPx-2 gene transcription at the tick stage, five 6-week-old male mice (Slac, China) were injected with RBCs infected with *B. microti.* Two days after injection, *Haemaphysalis longicornis* larval ticks were placed on the mice. A total of 54 engorged larval ticks were collected, which were cultivated in a 24°C incubator until these exuviated into nymphs. The nymph ticks were placed on the mice, engorged nymph ticks were then collected and cultivated to the adult phase. Next, 24 adult ticks were harvested for DNA extraction with a DNA extraction kit (Shanghai SBS Genetech, China), and the 18S RNA of *B. microti* was detected by qRT-PCR. Then, the 30 other adult ticks were divided into 10 groups for RNA extraction, the 10-group RNA for *BmTPx-2* transcription analysis.

### Ethics Statement

The present work was approved by the Animal Ethics Committee of Shanghai Veterinary Research Institute, Chinese Academy of Agricultural Sciences (CAAS) (No. SHVRI-Mo-0158). The protocol was approved by the Animal Care and Use Committee of the Shanghai Veterinary Research Institute, CAAS.

## Results

### Molecular Cloning of *BmTPx-2* and Multiple Sequence Alignment Analysis

**FIGURE 1:**
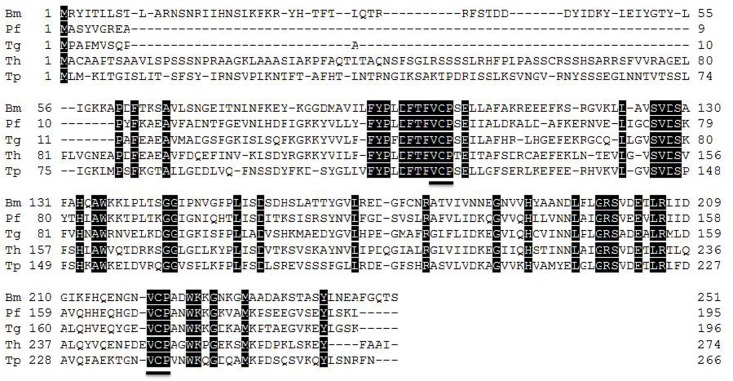
BmTPx-2 multiple sequence alignment analysis. *Babesia microti* (Bm: MF459654); *Plasmodium falciparum* (Pf: BAA97121.1); *Toxoplasma gondii* (Tg: AAG25678.2); *Tamarix hispida* (Th: AFH74407.1); *Theileria parva* Muguga (Tp: XP_765704.1). The two conserved VCP motifs are underlined.

The full-length cDNA of *BmTPx-2* was 826 bp, and the ORF length was 756 bp (GenBank Accession Number MF459654), encoding 251 amino acid residues without a signal peptide. The protein had a molecular weight of 28 kDa, and a pI of 8.231. BLASTp (NCBI) analysis determined that *BmTPx-2* belongs to the 2-Cys Prx2 family. Compared to other known 2-Cys Prxs, we found that *BmTPx-2* also contained two conserved Cys VCP motif residues at Cys^101^ and Cys^221^ (**Figure 1**). The black boxes with black letters indicate similar residues. The two conserved VCP motifs are underlined.

### Expression and Purification of the Recombinant BmTPx-2 Protein

**FIGURE 2:**
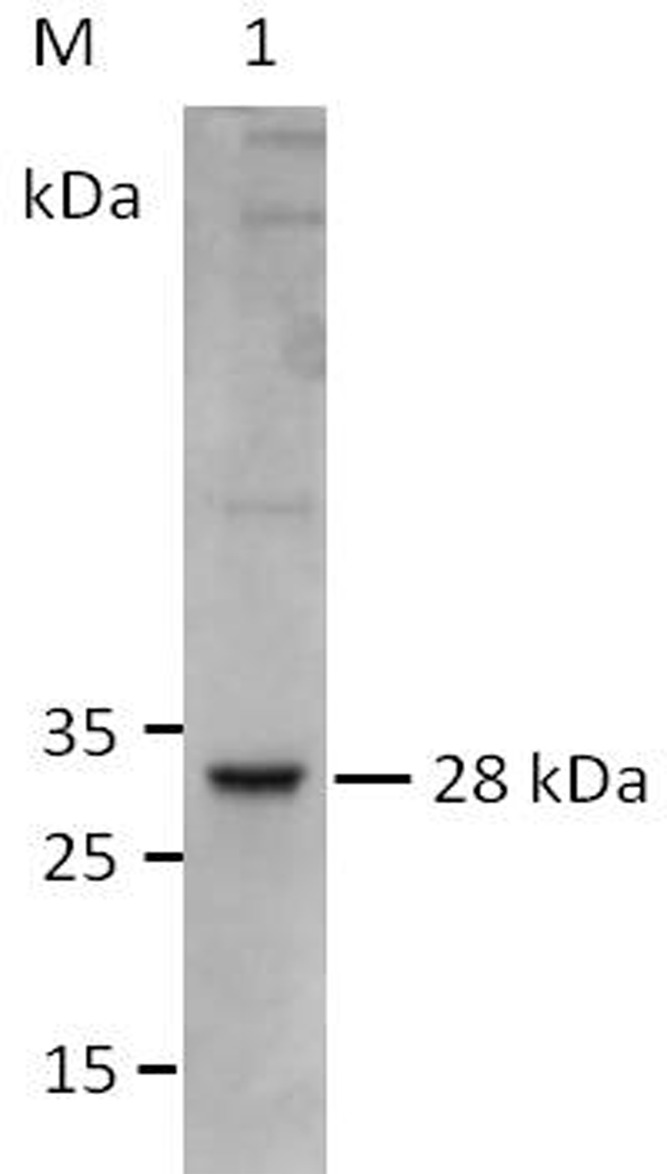
Expression and purification of recombinant BmTPx-2 protein. M, PageRuler prestained protein ladder; 1, rBmTPx-2 was expressed in *Escherichia coli* BL21 and purified with Ni-NTA agarose beads. Purified rBmTPx-2-His was approximately 28 kDa and was used in subsequent antibody production and antioxidant activity assays.

A fragment of the *BmTPx-2* ORF was amplified by PCR, and the product was cloned into a pET-28a vector and transformed into *E. coli* BL21(DE3). The fusion protein was expressed at 20°C for 8 h and purified using Ni-NTA agarose beads. The fusion and purification of the recombinant proteins was identified by SDS-PAGE and standard Coomassie brilliant blue staining. The results indicated that rBmTPx-2 had a molecular mass of about 28 kDa (**Figure 2**).

### Antioxidant Activity of the BmTPx-2 Protein

**FIGURE 3:**
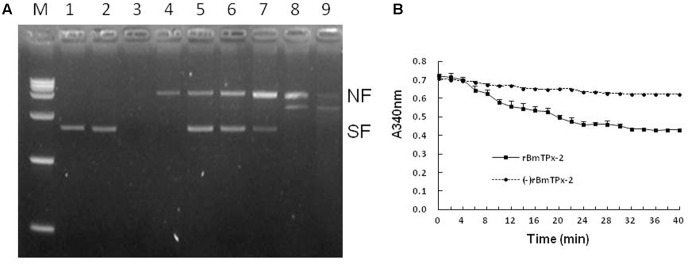
Antioxidant activity of the BmTPx-2 protein. **(A)** DNA protection experiment analyzed by MFO system. M, DNA marker; (1) DNA+ H_2_O; (2) DNA+ DTT; (3) DNA+ FeCl_3_; (4) DNA+MFO; (5) 335 μg/mL rBmTPx-2+MFO+DNA; (6) 167 μg/mL rBmTPx-2+MFO+DNA; (7) 83 μg/mL rBmTPx-2+MFO+DNA; (8) 55 μg/mL rBmTPx-2+MFO+DNA; (9) 42 μg/mL rBmTPx-2+MFO+DNA. The nicked form (NF) and supercoiled form (SF) of the plasmids are indicated on the right and the triangle shows the increasing concentration of the recombinant proteins. **(B)** Antioxidant function of identification by using a Trx-TrxR assay system. The solid line indicates the antioxidant function of rBmTPx-2; the dotted line indicates the absence of rBmTPx-2. NADPH oxidation was monitored as the decrease in **(A)** 340 nm. The Data are presented as the means of three independent experiments.

Both FeCl_3_ and DTT of the MFO system produced hydroxyl radicals that can damage the plasmid, and the molecular weight of the nicked plasmid apparently increased (**Figure 3A**). However, the presence of rBmTPx-2 protected the plasmid from hydroxyl radical-induced damage. When the concentration of rBmTPx-2 was 335 μg/mL, about half of the DNA was undamaged, but when the concentration of BmTPx-2 was reduced to 41 μg/mL, nearly all the DNA was damaged. These findings indicated an inverse relationship between the concentration of rBmTPx-2 and the quantity of the nicked plasmid. These results suggest that the antioxidant activity of rBmTPx-2 was concentration-dependent (**Figure 3A**).

The Trx-TrxR assay system showed a decrease in the absorbance at a wavelength of 340 nm; however, the degree of the decline was not significant, in which after 40 min, the absorbance decreased about 0.32. The absorbance of the negative control group decreased to about 0.08 compared to that of the BmTPx-2 group (**Figure 3B**).

### Western Blot Analysis of the Native BmTPx-2 Protein of Parasites

**FIGURE 4:**
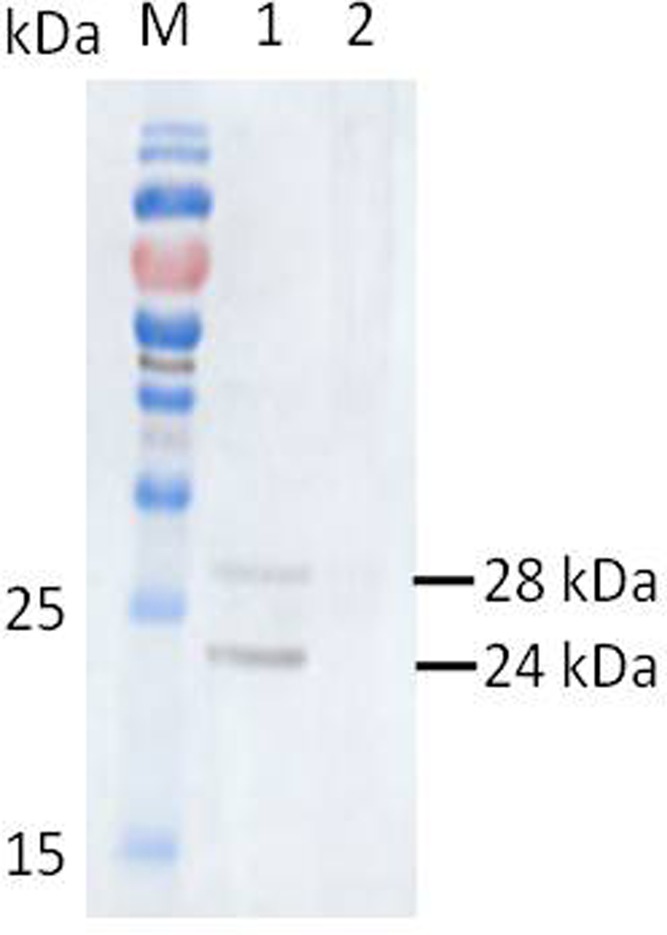
Western blot analysis for native protein of parasites. M, PageRuler prestained protein ladder; (1) RBCs from mice infected with *B. microti*; (2) RBCs from mice uninfected with *B. microti*.

*Babesia microti* native BmTPx-2 protein extracted from RBCs of mice infected with *B. microti* was detected using an anti-BmTPx-2 mouse antiserum, whereas the negative control showed no specific bands (**Figure 4**). The results demonstrated that the molecular weight of BmTPx-2 of *B. microti* were about 28 or 24 kDa.

### Transcription Analysis at the Erythrocyte Stages and Tick Stages

**FIGURE 5:**
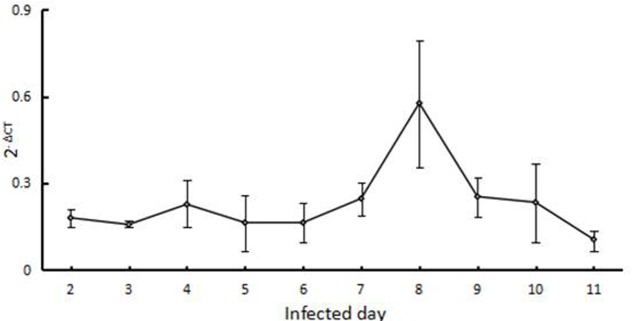
Transcription analysis at the erythrocyte stage. x-axis refers to the day of infection of mouse RBCs with *B. microti*; the y-axis refers to the relative BmTPx-2 expression level in mouse RBCs at different days after infection with *B. microti* by using the 2^-ΔΔ*C*_T_^ mean.

The level of *BmTPx-2* mRNA expression in erythrocytes was maintained at a relatively stable level from the 2nd to 7th day (**Figure 5**). BmTPx-2 expression peaked on the 8th day and then decreased on the 9th day. On the other hand, no *B. microti 18S* DNA was detected in 24 adult ticks by using RT-PCR, and we also could not detect the *BmTPx-2* transcription of *B. microti* among 10 adult RNA.

## Discussion

Prx plays important roles in protecting parasites from damage induced by ROS in host erythrocytes, as well as in *B. microti* (Tanaka et al., 2009; Robinson et al., 2010). Prxs were initially discovered in 1989 to have an ability to resolve organic peroxide cumene hydroperoxide. Although not all of the role of Prxs have been confirmed in parasites, its major function involves thioredoxin-dependent peroxidase activity (Gretes et al., 2012; Angelucci et al., 2016). In the present study, we obtained the *BmTPx-2* full-length cDNA, which comprised a 756-bp long ORF that encodes a 251-amino acid protein without a signal peptide, with a predicted molecular weight of 28 kDa. BLASTp analysis revealed that BmTPx-2 belongs to the 2-Cys Prx family, contained two VCP motifs, and the conserved cysteine residues were Cys^101^ and Cys^221^, which is similar to that of the other 2-Cys Prxs. In our previous study, we described the identification and functional study of a novel 2-Cys peroxiredoxin (BmTPx-1) of *B. microti* (Zhang et al., 2016). In this study, the BLASTp results showed that BmTPx-2 had 38% sequence similarity with BmTPx-1, 48% with of Peroxiredoxin-2 (Prx2) of *Babesia* sp. Xinjiang (ORM40258.1), 42% with Thioredoxin peroxidase 2 (TPx-2) of *P. falciparum* 3D7 (XP_001350554.1). The BmTPx-2 showed highest homology with Prx2 (TPx-2), so we named this novel gene as BmTPx-2.

To explore the enzymatic activity of BmTPx-2, we prepared a recombinant BmTPx-2 (rBmTPx-2) protein, and its antioxidant activity was evaluated using an MFO system, which showed that rBmTPx-2 was capable of protecting plasmids from damage, thereby indicative of BmTPx-2 antioxidant activity. BmTPx-2 possibly acts as an antioxidant enzyme that catalyzes hydrogen peroxide in *B. microti*. The main function of Prxs in parasites involves resolving H_2_O_2_ with Trx, TrxR, and NADPH. Prxs can eliminate hydrogen peroxide with thioredoxin by using thioredoxin as the electron donor (Rhee et al., 2005). Monitoring the oxidation of NADPH suggested that BmTPx-2 plays functions as a peroxide reductase in *B. microti*. Furthermore, western blot analysis revealed that anti-BmTPx-2 mice serum can recognize the native BmTPx-2 protein of *B. microti*. The molecular weight of the native BmTPx-2 protein of *B. microti* were about 24 or 28 kDa. This discrepancy may be due to the different modification used in *B. microti*. Moreover, the appearance of an extra band in Lane 1 of **Figure 4** may be due to the antiserum comprising polyclonal antibodies; therefore, the antiserum may recognize other proteins of *B. microti* besides BmTPx-2. BmTPx-2 transcription during the erythrocyte stage increased from the 2nd to the 7th day and then peaked on the 8th day, thereby suggesting maximal antioxidant response before weakening from the 9th to the 11th day. We could not detect the 18S DNA and *BmTPx-2* RNA transcription at the tick stages. This may be due to two reasons: (i) the number of *B. microti* that was ingested by the ticks might have extremely low in that the resulting 18S DNA and *BmTPx-2* RNA levels were below the detection limit of the assay; (ii) it is possible that upon placing the ticks on healthy mice, the *B. microti* pathogens might have been excluded from the salivary glands of ticks. Future studies are necessary to confirm these hypotheses.

Recent studies knockout and RNAi experiments have determined that Prxs are crucial to the survival and virulence of parasites. Kimura et al. (2013) found that the disruption of the *Prx1* gene of *P. falciparum* induced hypersensitivity to heat stress. Usui et al. (2015) demonstrated that TPx-2-KO led to death of *Plasmodium berghei* at the liver stage. To the best of our knowledge, this is the first report that provides evidence that TPx-2 has an antioxidant function against the toxicity of ROS. However, BmTPx-2 is just one of the several Prxs in *B. microti*, and we believe that a complex relationship among these Prxs may exist, which will be the focus of our future research. Additionally, antioxidant Prxs play other functions such as molecular chaperones, sensors of peroxides, and modulators of inflammatory response (Angelucci et al., 2016). Therefore, in future studies, we will investigate whether BmTPx-2 is involved in these additional functions in *B. microti*. In conclusion, this study provides foundational basis of the role of BmTPx-2 in *B. microti* and may be a potential target in treating babesiosis infections.

## Author Contributions

XH and HZ conducted gene identification and functional studies. XH wrote the draft of the manuscript. JZ and HZ corrected the manuscript. ZW, HG, JC, and YZ analyzed the results. All authors have read and approved the final manuscript.

## Conflict of Interest Statement

The authors declare that the research was conducted in the absence of any commercial or financial relationships that could be construed as a potential conflict of interest.
